# Odontogenic Differentiation of Human Dental Pulp Stem Cells on Hydrogel Scaffolds Derived from Decellularized Bone Extracellular Matrix and Collagen Type I

**DOI:** 10.1371/journal.pone.0148225

**Published:** 2016-02-16

**Authors:** Francesco Paduano, Massimo Marrelli, Lisa J. White, Kevin M. Shakesheff, Marco Tatullo

**Affiliations:** 1 Tecnologica Research Institute, Biomedical Section, Crotone, Italy; 2 Unit of Maxillofacial Surgery, Calabrodental, Crotone, Italy; 3 School of Pharmacy, University of Nottingham, Nottingham, NG7 2RD, United Kingdom; Texas A&M University Baylor College of Dentistry, UNITED STATES

## Abstract

**Objectives:**

The aim of this study was to evaluate the level of odontogenic differentiation of dental pulp stem cells (DPSCs) on hydrogel scaffolds derived from bone extracellular matrix (bECM) in comparison to those seeded on collagen I (Col-I), one of the main components of dental pulp ECM.

**Methods:**

DPSCs isolated from human third molars were characterized for surface marker expression and odontogenic potential prior to seeding into bECM or Col-I hydrogel scaffolds. The cells were then seeded onto bECM and Col-I hydrogel scaffolds and cultured under basal conditions or with odontogenic and growth factor (GF) supplements. DPSCs cultivated on tissue culture polystyrene (TCPS) with and without supplements were used as controls. Gene expression of dentin sialophosphoprotein (DSPP), dentin matrix protein 1 (DMP-1) and matrix extracellular phosphoglycoprotein (MEPE) was evaluated by quantitative reverse transcription-polymerase chain reaction (qRT-PCR) and mineral deposition was observed by Von Kossa staining.

**Results:**

When DPSCs were cultured on bECM hydrogels, the mRNA expression levels of DSPP, DMP-1 and MEPE genes were significantly upregulated with respect to those cultured on Col-I scaffolds or TCPS in the absence of extra odontogenic inducers. In addition, more mineral deposition was observed on bECM hydrogel scaffolds as demonstrated by Von Kossa staining. Moreover, DSPP, DMP-1 and MEPE mRNA expressions of DPSCs cultured on bECM hydrogels were further upregulated by the addition of GFs or osteo/odontogenic medium compared to Col-I treated cells in the same culture conditions.

**Significance:**

These results demonstrate the potential of the bECM hydrogel scaffolds to stimulate odontogenic differentiation of DPSCs.

## Introduction

Currently, the standard clinical treatment for deep dental caries reaching the pulp is endodontic therapy that includes cleaning of the pulp chamber and the replacement of the necrotic pulp tissue with bio-inert materials [1]. This treatment has several limitations with the teeth losing their vitality and sensitivity and becoming susceptible to secondary infections and to post-operative fractures [2]. Regenerative approaches offer a promising strategy to achieve dentin-pulp repair [3]. In the field of regenerative dentistry, researchers have explored new methodologies for the treatment of injured dental structures through the development of novel dentin-pulp tissue based on the combination of mesenchymal stem cells (MSCs) of oral origin, growth factors and scaffolds [3].

The identification of mesenchymal stem cells from several dental tissues such as dental pulp [4], periodontal ligament [5], root apical papilla [6], exfoliated deciduous teeth [7] and periapical cyst [8–10] has made pulp regeneration a realistic clinical possibility. Dental pulp stem cells (DPSCs) are particularly interesting because they possess clonogenic ability, rapid proliferation rate and are multipotent [4, 10–13]. Another characteristic of DPSCs is their capacity to differentiate into odontoblastic cell lineages [12, 14]. Gronthos *et al*. demonstrated that it was possible to induce dentin formation from DPSCs placed subcutaneously in immunocompromised mice when transplanted in conjunction with HA/TCP powder [4]. Moreover, several studies have demonstrated that DPSCs are a promising cell source for dental tissue regeneration due to their ability to differentiate into odontoblast-like cells *in vitro* and to form dentin-pulp structures *in vivo* when seeded on scaffolds [15–17].

A wide variety of both synthetic and natural scaffolds have been used to support the differentiation of DPSCs towards an odontoblastic-like phenotype *in vitro* and towards the regeneration of pulp-dentin tissues *in vivo* [3, 18–22]. Given the role of collagen in the extracellular matrix (ECM) of dental pulp and dentin, this material has attracted interest as a scaffold for dental pulp tissue engineering [23]. Collagen I scaffolds accelerated odontogenic differentiation and mineralization of stem cells from exfoliated deciduous teeth (SHED) [24]. Moreover, a scaffold composed of collagen type I (Col-I) has been used in combination with human DPSCs and growth factors such as DMP-1 to form dental pulp-like tissues in preclinical studies [25]. Furthermore, by using collagen scaffolds with DMP-1, investigators have obtained differentiation of DPSCs into odontoblasts-like cells and the formation of dentin-like tissues in a rat model [26].

Recently, several studies have used materials that mimic aspects of the architecture of natural dental ECM for the regeneration of damaged dentin/pulp tissues. Qu *et al*. demonstrated that gelatin/bioactive glass hybrid scaffolds provided an excellent environment for the odontogenic differentiation of DPSCs [27]. Moreover, Ravindran *et al*. developed an ECM scaffold composed of collagen I/chitosan that was able to induce odontogenic differentiation of DPSCs and to form dental pulp-like tissue when implanted subcutaneously in mice [28]. Recently, Zhang *et al*. have demonstrated that scaffolds derived from small intestinal submucosa (SIS) promoted odontogenic differentiation of DPSCs [29].

A recent study by Sawkins *et al*. demonstrated that hydrogel scaffolds derived from decellularized and demineralized bovine bone (bECM) promoted proliferation of mouse primary calvarial cells (mPCs) compared to Col-I hydrogel scaffolds [30].

Further investigations with bECM hydrogel scaffolds have demonstrated the utility of bone derived ECM hydrogels as a vehicle for delivering osteogenic factors in an organotypic chick femur culture model [31, 32]. More recently, bECM hydrogel scaffolds have provided *in vivo* tissue mineralization and bone formation [33].

Here, we evaluated the effect of bECM hydrogel scaffolds on the odontogenic differentiation of DPSCs, in presence or absence of extra-odontogenic inducers or growth factors, by evaluating changes in expression of genes involved in odontogenesis.

## Materials and Methods

### Cell isolation and culture

Human-impacted third molars were obtained with written informed consent from Calabrodental Dental Clinic, Crotone, Italy. Patients were all volunteers collaborators of Calabrodental, undergone to dental extractions for clinical reasons. The Ethical Committee at the Calabrodental Dental Clinic on 2013 approved the use of human third molars (ethical agreement number CBD-008/TRI/2015). All clinical investigations have been conducted according to the principles expressed in the Declaration of Helsinki.

To isolate DPSCs, normal human third molars were obtained from four young healthy patients (age 20–25). The teeth were washed in PBS containing 100 U/ml penicillin and 100 mg/ml streptomycin and transferred to the cell culture laboratory where mesenchymal stem cells from the pulp tissues were isolated according to a previously published method [4]. Briefly, pulp tissues were washed several times with PBS (Invitrogen) and cut into small pieces with a scalpel under sterile conditions. The pulps were extracted and analysed in Italy and were not pooled. Subsequently, the pulp tissue was digested with an enzyme solution that consisted of 3 mg/ml type I collagenase (Sigma) and 4 mg/ml dispase (Sigma) in Hank’s Balanced Salt Solution (HBSS, Invitrogen) for 1h at 37°C with regular agitation. The digest was added to 3 mL of alpha-minimal essential medium (α-MEM, Gibco) supplemented with 10% FBS (Gibco), and centrifuged at 300 g for 5 minutes. After centrifugation, the pellet was suspended in fresh α-MEM, seeded in culture dishes and incubated in 5% CO_2_ at 37°C. In order to verify the odontogenic capability, the extracted DPSCs at passage 4 were cultured on tissue culture polystyrene (TCPS) for 3 weeks in osteo/odontogenic medium, consisting of αMEM supplemented with 10% FBS, 50 μg/ml ascorbic acid, 5 mM β-Glycerophosphate and 10 nM dexamethasone.

### Phenotypic analysis of DPSCs by flow cytometry

The expression of MSC cell-surface markers in DPSCs was determined by FACS analysis. Cells were prepared as single cell suspensions and aliquoted at 0.5 x 10^6^ cells into FACS tubes. Subsequently, the cells were resuspended in 3% BSA blocking buffer and incubated for 30 minutes in the dark with isothiocyanate-conjugated anti-CD73 (CD73-FITC), phycoerythrin-conjugated anti-CD90 (CD90-PE), allophycocyanin-conjugated anti-CD105 (CD105-APC), PE-conjugated anti-CD13 (CD13-PE), APC-conjugated anti-CD29 (CD29-APC), FITC-conjugated anti-CD44 (CD44-FITC), PE-conjugated anti-CD146 (CD146-PE), allophycocyanin-H7-conjugated anti-CD45 (CD45-APC-H7) and PE-conjugated anti-HLA-DR (CD13-PE). All antibodies were purchased from BD (BD Pharmigen). Nonspecific background was evaluated by parallel staining with different IgG isotypes coupled to FITC, PE, APC and APC-H7 fluorochromes (BD Pharmigen). At the end of the incubation period, cells were washed twice with PBS and resuspended in 500 μl of PBS and 1% PBS. All flow cytometry measurements were made using a NAVIOS instrument (Beckman Coulter) and the data were analysed by Kaluza 1.3 program (Beckman Coulter).

### Preparation of bECM and Col-I hydrogel scaffolds

bECM derived from demineralized and decellularized bovine bone was prepared using a previously described method [30–32, 34]. Briefly, bovine tibias were harvested from cattle, aged 12–24 months, slaughtered by an EU certified butcher (J Broomhall Ltd., Gloucestershire, UK) and then segmented into cancellous and cortical groups, with the cancellous group used in this study. Cancellous segments were cleaned of residual tissue and washed with phosphate buffered saline (PBS) containing 0.1% w/v Gentamicin (Invitrogen) and then frozen in liquid nitrogen and sectioned to produce fragments no greater than 4 x 4 x 4 mm which were ground in a commercial coffee mill (Krups F203). The resultant granules were demineralized under agitation, 300 rpm, in 0.5 N HCl (25 ml per gram of bone) at room temperature for 24 hours. The lipid in the demineralized powder was then extracted with a 1:1 mixture of chloroform (Fisher Scientific) and methanol (Fisher Scientific) for 1 hr. The demineralized powder was then decellularized in a solution of 0.05% Trypsin (Sigma-Aldrich) and 0.02% Ethylenediaminetetraacetic acid (EDTA) (Sigma-Aldrich) at 37°C and 5% CO_2_ under continuous agitation for 24 hours. The resultant material, referred to as as bone decellularized matrix (bECM) was rinsed in PBS supplemented with 1% w/v penicillin/streptomycin under continuous agitation for 24 hours at 4°C to remove residual cellular material. The bECM was then snap frozen, lyophilised overnight and stored at -20°C until required.

A pepsin digestion and solubilisation technique was used to produce bECM digests: 1 g dry bECM was mixed with 100 mg pepsin in 100 ml of 0.01 N HCl. The suspension was mixed on a stirrer plate at room temperature for 96 hours, until no visible pieces of matrix remained. The resultant bECM digest was aliquoted and stored at -20°C until required. Gelation of the bECM occurred by neutralizing the salt concentration and pH of the pepsin digest at 4°C (to form a pre-gel solution) which then formed a hydrogel upon warming to 37°C. Thus bECM hydrogel scaffolds were formed by mixing 0.1 N NaOH (1/10 of the volume of pre-gel solution) and 10X PBS pH 7.4 (1/9 of the volume of pre-gel solution) and by then diluting to the desired final bECM concentration with 1X PBS at 4°C [30]. The pre-gels were transferred to dishes and then placed in a non-humidified incubator heated to 37°C for gelation to occur. Once the hydrogel scaffolds had formed, dishes were washed with PBS and then with α-MEM before cells were added. bECM hydrogels at concentrations of 3, 4, 6, and 8 mg/ml were prepared. Rat-tail collagen type I (Col-I) was purchased from Corning and Col-I hydrogels (3, 4, 6 and 8 mg/ml) were prepared with the same protocol used for bECM.

### Cell culture and seeding onto bECM or Col-I hydrogel scaffolds

DPSCs were seeded onto the surface of the hydrogel scaffolds at a density of 0.5 x 10^6^/ml and cultured for 3 weeks. The DPSCs/scaffold constructs were cultured in basal medium, osteo/odontogenic medium or medium supplemented with growth factors. Basal medium consisted of α-MEM supplemented with 10% FBS. For the odontogenic differentiation of DPSCs basal medium was supplemented with 50 μg/ml ascorbic acid, 5 mM β-Glycerophosphate and 10 nM dexamethasone. Medium supplemented with GFs consists of α-MEM supplemented with 10% FBS, 40 ng/ml Fibroblast Growth Factor basic (FGFb, Sigma) and 20 ng/ml Epidermal Growth Factor (EGF, Invitrogen). All DPSCs/scaffold constructs were cultured at 37°C with 5% CO_2_ for 3 weeks and the culture medium was changed every two days.

### Live/Dead Staining and confocal microscopy

DPSCs were seeded on the bECM scaffolds as described previously. At two and three weeks post seeding, live/dead staining was performed on bECM hydrogel scaffolds. A staining solution was prepared in PBS containing 20 μg/ml propidium iodide (Sigma-Aldrich) and 1 μg/ml fluorescein diacetate (Calbiochem). 100 μl of the staining solution was added to each scaffold and incubated at room temperature for 15 minutes before visualization using a Leica LCS confocal microscope (Leica; TCS SP5). Live cells stained green and dead cells stained red.

### DPSC isolation from bECM and Col-I hydrogel scaffolds for RNA extraction

bECM and Col-I hydrogel scaffolds containing cells were washed twice in PBS (Gibco), and digested with an enzyme solution that consisted of 3 mg/ml type I collagenase (Sigma) and 4 mg/ml dispase (Sigma) in Hank’s Balanced Salt Solution (HBSS, Invitrogen) for 1h at 37°C. Recovered cells were centrifuged and washed twice in PBS, then total RNA was extracted from DPSCs using the Purelink™ RNA mini kit (Applied Biosystems) as described by the supplier, and the extracted RNA was quantified using a Multiskan Go spectrophotometer (Thermo Scientific). TCPSs containing cells were washed in PBS and digested with trypsin. Recovered cells were centrifuged and washed in PBS, then total RNA was extracted from DPSCs as described above.

### Quantitative real-time PCR (qRT-PCR)

To quantify relative gene expression levels, 500 ng of each RNA was reverse-transcribed using M-MuLV reverse transcriptase (High Capacity RNA-to-cDNA Kit, Applied Biosystems) in a 20 μl volume containing 0.2 μg Oligo(dT), 400 μmol/l dNTPs and buffers according the manufacturer’s instructions. mRNA levels for dentin sialophosphoprotein (DSPP) and dentin matrix acidic phosphoprotein (DMP-1), as odontogenic differentiation marker genes, were measured by real-time quantitative reverse transcriptase-polymerase chain reaction (qRT-PCR). The 10 μL reaction contained 0.5 μL cDNA from each sample mixed with 5 μl power SYBR green PCR Master Mix (Applied Biosystems), 0.25 μl of each sense and antisense primers and 4 μl RNase/DNase-free water. The real-time qPCR reactions were performed in the thermal cycler Pikoreal 96 instrument (Thermo Scientific). The real-time qPCR conditions for DSPP and DMP-1 were as follows: an initial denaturation step at 95°C for 10 min and then 40 cycles of 10 s at 95°C and 1 min at 60°C. Amplification conditions for MEPE were: an initial denaturation step at 95°C for 10 min and then 45 cycles of 10 minute at 95°C, 1 min at 55°C and 1 min at 72°C [35].

Real-time efficiencies were calculated from the cycle threshold (Ct) curves obtained for the amplification of the cDNA samples (serial ten-fold dilutions: 1, 0.1, 0.01 and 0.001). The specificity of the qPCR products was evaluated by melting curve analysis. The expression levels of the target genes were obtained from the threshold PCR cycle-values (Ct). Relative expression levels were calculated using the comparative Ct method (ΔΔCt) and by normalising to hypoxanthine phosphoribosyltransferase (HRPT). The primers for dentin sialophosphoprotein (DSPP), dentin matrix acid phosphoprotein-1 (DMP-1), matrix extracellular phosphoglycoprotein (MEPE) and hypoxanthine phosphoribosyltransferase (HPRT) are listed in Table 1.

**Table 1:** Primer sequences used in qRT-PCR analysis.

Gene	Sequence (5’-3’)	NCBI Accession Number
**DSPP**	**Forward:** CTGTTGGGAAGAGCCAAGATAAG	NM_014208.3
	**Reverse:** CCAAGATCATTCCATGTTGTCCT	
**DMP-1**	**Forward:** GTGAGTGAGTCCAGGGGAGATAA	NM_004407.3
	**Reverse:** TTTTGAGTGGGAGAGTGTGTGC	
**MEPE**	**Forward:** CCCTGGAAGAGAAGGAAACAGA	NM_001184694.2
	**Reverse:** TGAAACTCAACCTTCCCTTGGT	
**HPRT**	**Forward:** TGACACTGGCAAAACAATGCA	NM_000194.2
	**Reverse:** GGTCCTTTTCACCAGCAAGCT	

DSPP, dentin sialophosphoprotein; DMP-1, dentin matrix acid phosphoprotein-1; MEPE, matrix extracellular phosphoglycoprotein; HPRT, hypoxanthine phosphoribosyltransferase.

### Von Kossa staining

After 3 weeks of culture, the DPSCs/bECM constructs were rinsed with PBS, and then fixed with 10% (w/v) formalin. A Von Kossa method for calcium Kit was used to stain calcium deposits according to the supplier’s method (Bio-Optica).

### Statistical analysis

All statistical analyses were performed using GraphPad Prism statistical program (Graphpad Software, Inc). qRT-PCR were analysed using an unpaired two-tailed Student’s t-test. Significance for all statistical analyses was defined as *P* < 0.05 (*) and *P* < 0.01 (**). All graphed values represent the means ± standard deviations (SDs) from three separate experiments.

## Results

### Phenotypic analysis of cell-surface markers of DPSCs and evaluation of gene expression during odontogenic differentiation

**Fig 1:**
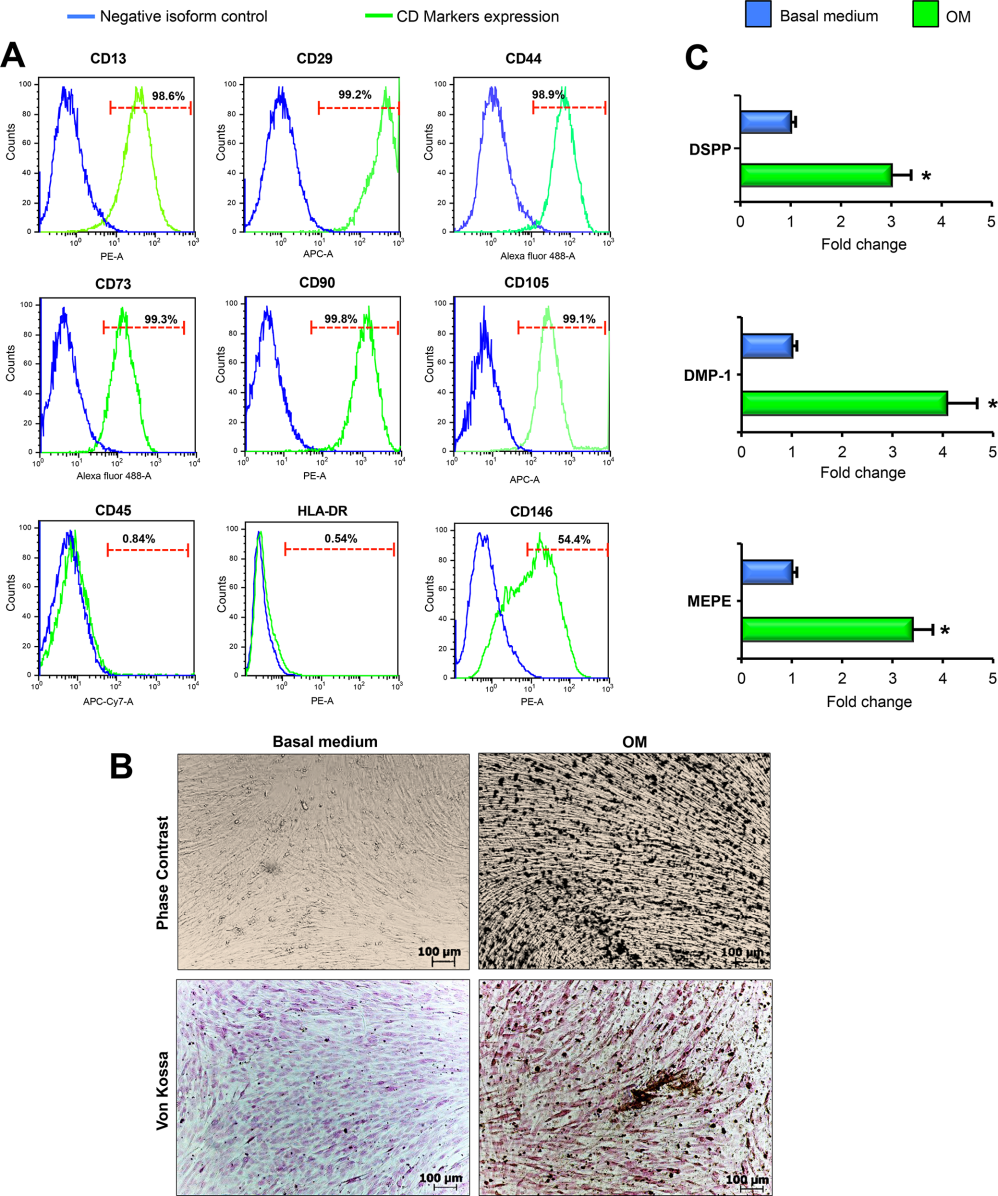
MSCs surface markers and odontogenic genes expression of DPSCs cultured on TCPS (tissue culture polystyrene) prior to seeding into bECM hydrogel scaffolds. (A) Immunophenotype analysis of CD13, CD29, CD44, CD73, CD90, CD105, CD146, CD45 and HLA-DR expression in DPSCs by flow cytometry. The green histograms represent the cell count for the specific antibody, and the blue histograms represent the fluorescence of the negative control. The percentage of cells positive for each antigen is shown in the figure. (B) Phase contrast and Von Kossa staining of human DPSCs cultured on TCPS and maintained in basal or osteo/odontogenic medium for 3 weeks (dark color indicates mineral nodules), scale bar: 100 μm. OM: osteo/odontogenic medium. (C) qRT-PCR at 3 weeks for odontogenic markers DSPP, DMP-1 and MEPE of DPSCs seeded on TCPS cultured in basal medium compared to those cultured in osteo/odontogenic medium. The results are presented as the fold increase (2^-(ΔΔCT)^) with respect to the level expressed in DPSCs cultured in basal medium. All values are expressed as the means ± SDs and were normalised to HPRT expression levels, comparison by unpaired two-tailed Student’s t-test. *P < 0.05. OM: osteo/odontogenic medium.

Prior to seeding DPSCs onto bECM and Col-I hydrogel scaffolds, flow cytometry was performed to confirm the mesenchymal surface markers expression of the cells. DPSCs were positive for MSCs markers CD13, CD29, CD44, CD73, CD90, CD105, CD146 and negative for the hematopoietic markers CD45 and HLA-DR (Fig 1A). After 3 weeks of odontogenic induction of DPSCs, a mass of mineralized deposits was formed (Fig 1B). No mineralisation was evident without induction. Von Kossa staining also revealed mineral deposition in DPSCs after 3 weeks of culture in osteo/odontogenic medium, while calcium deposition was absent in cells cultured in basal medium (Fig 1B). Expression levels of the major odontogenic-specific genes DSPP, DMP-1 and MEPE were significantly higher under osteo/odontogenic medium conditions (Fig 1C). These data showed the odontogenic potential of DPSCs.

### Cell morphology observation on bECM and Col-I hydrogel scaffolds

**Fig 2:**
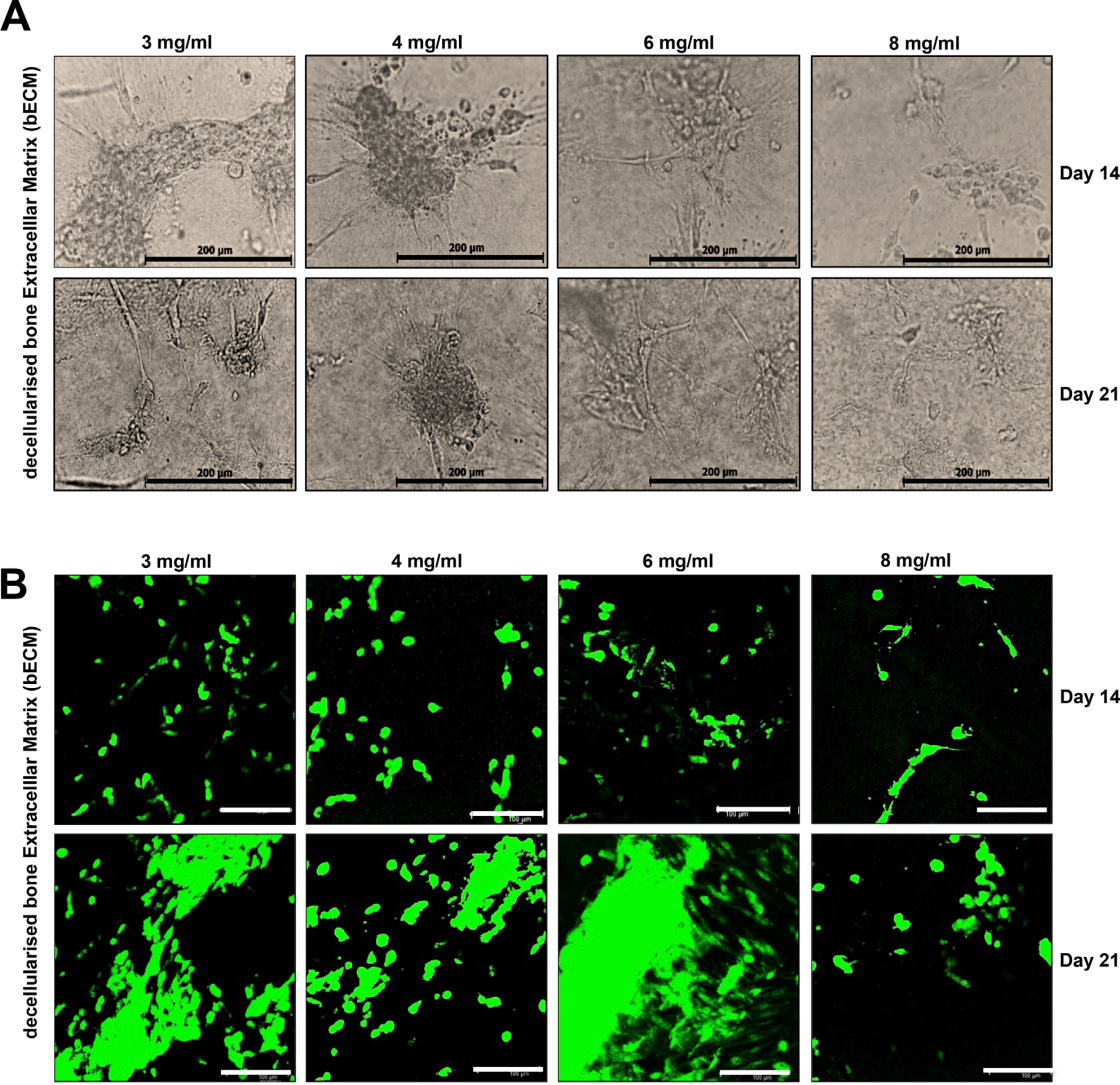
Cell morphology and viability of DPSCs in bECM scaffolds *in vitro*. (A) Representative images of DPSCs seeded on bECM scaffolds (3, 4, 6, 8 mg/ml) at day 14 and 21. DPSCs proliferate, elongate and form clusters, scale bar: 100 μm. (B) DPSCs remained viable after 14 and 21 days of culture on 3, 4, 6, 8 mg/ml bECM hydrogel scaffolds. Viable cells stained green and dead cells stained red after CMFDA/PI staining. Cells were observed under fluorescence confocal microscope, scale bar: 100 μm.

DPSCs were seeded onto bECM and Col-I hydrogel scaffolds (3, 4, 6, 8 mg/ml), and then the morphology and distribution of cells were observed. DPSCs grew in distinct independent clusters or colonies on 3, 4, 6, 8 mg/ml bECM hydrogel scaffolds compared to TCPS (Fig 2A and S1 Fig). A similar distribution of cells was also observed on Col-I hydrogel scaffolds. Cells seeded on 3, 4, 6, 8 mg/ml bECM hydrogels remained viable during initial cell seeding (S2 Fig) and covered the scaffolds after 14 and 21 days *in vitro* culture as observed by widespread cytoplasmic staining with cell tracker green (Fig 2B), indicating that DPSCs were viable on bECM hydrogel scaffolds; similar results were obtained with Col-I hydrogel scaffolds (data not shown).

### bECM hydrogel scaffolds spontaneously upregulate odontogenic genes of DPSCs in basal culture conditions

**Fig 3:**
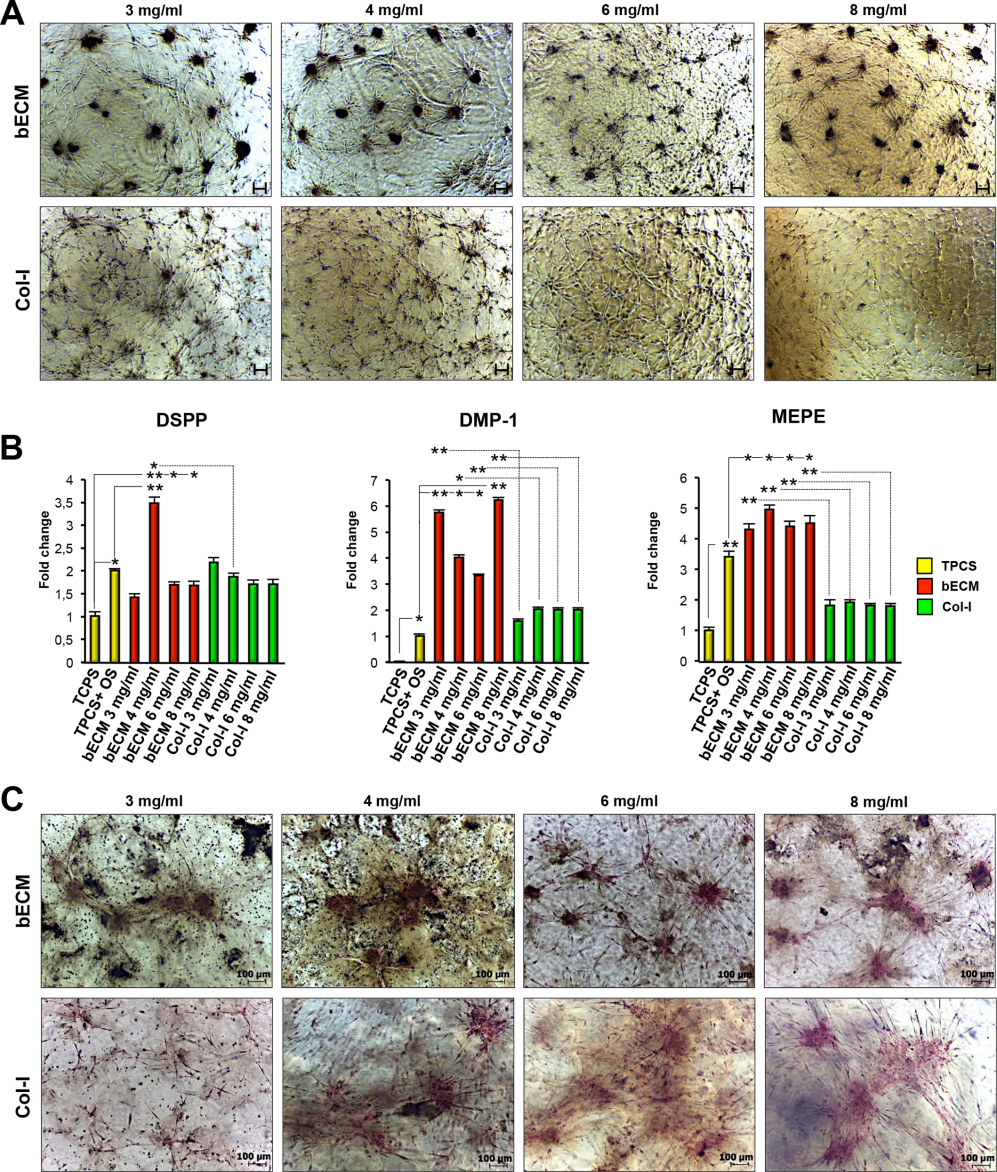
Cell morphology, mineralization and relative mRNA expression of odontogenic genes in DPSCs cultured on bECM or Col-I hydrogel scaffolds in normal culture conditions. (A) Representative images of human DPSCs cultured on bECM hydrogel scaffolds (3, 4, 6, 8 mg/ml) or Col-I hydrogel scaffolds (3, 4, 6, 8 mg/ml) in basal medium for 3 weeks, scale bar: 100 μm. (B) qRT-PCR at 3 weeks for odontogenic markers DSPP, DMP-1 and MEPE of DPSCs seeded on bECM (3, 4, 6, 8 mg/ml), Col-I (3, 4, 6, 8 mg/ml) hydrogel scaffolds or TCPS cultured in basal medium compared to those seeded on TCPS in presence of osteo/odontogenic medium (OM). The results are presented as the fold increase (2^-(ΔΔCT)^) with respect to the level expressed in DPSCs seeded on TCPS cultured in basal medium. All values are expressed as the means ± SDs and were normalised to HPRT expression levels, comparison by unpaired two-tailed Student’s t-test. *P < 0.05, **P < 0.01. (C) Von Kossa staining of human DPSCs cultured on bECM (3, 4, 6, 8 mg/ml) and Col-I (3, 4, 6, 8 mg/ml) hydrogel scaffolds in basal medium for 3 weeks. Black color indicates mineral deposition. Scale bar: 100 μm.

To verify if the bECM hydrogel scaffolds influenced the odontogenic differentiation of DPSCs, cells were cultured in basal medium on the surface of 3, 4, 6, 8 mg/ml bECM or Col-I hydrogel scaffolds for 3 weeks (Fig 3A). Additionally, DPSCs were seeded on TCPS with basal medium or osteo/odontogenic medium. The mRNA expression levels of DSPP, DMP-1 and MEPE, three specific markers of odontogenic differentiation, were highly induced in both bECM and Col-I hydrogel scaffold cultures compared to TCPS, indicating the suitability of both hydrogel scaffolds for odontogenic differentiation of DPSCs (Fig 3B). Furthermore, the mRNA expression level of DSPP was significantly greater in DPSCs seeded on the 4 mg/ml bECM hydrogel scaffold compared to the 4 mg/ml Col-I scaffold or on TCPS in presence of osteo/odontogenic medium. There were no statistically significant differences in DSPP gene expression between DPSCs seeded on 3, 4, 6, 8 mg/ml Col-I hydrogel scaffolds compared to DPSCs cultured on TCPS with osteo/odontogenic medium.

The stem cell-scaffold constructs were also screened for the expression of DMP-1 and MEPE using qRT-PCR. The mRNA expression levels of DMP-1 and MEPE were considerably higher in DPSCs seeded on 3, 4, 6, 8 mg/ml bECM hydrogel scaffolds compared to those seeded on Col-I hydrogel scaffolds at the same concentrations or on TCPS in presence of osteo/odontogenic medium (Fig 3B). There were no significant differences in DMP-1 gene expression between DPSCs seeded on 3, 4, 6, 8 mg/ml Col-I hydrogel scaffolds compared to TCPS-cultured cells in medium containing odontogenic inducers. In addition, DPSCs seeded on TCPS and cultured in osteo/odontogenic medium expressed higher levels of MEPE compared to Col-I hydrogel scaffolds.

The mineralization on hydrogel scaffolds was examined by using Von Kossa staining. Results revealed substantial levels of mineral deposition (black color) after 3 weeks of culture on both types of hydrogel scaffolds (Fig 3C and S3 Fig). However, at the same culture times, there was more mineral generated on bECM than on Col-I hydrogel scaffolds.

Collectively, these results demonstrate that the combination of bECM hydrogel scaffolds with DPSCs is sufficient to induce odontogenic differentiation without requiring the additional odontogenic factors. Consequently, bECM hydrogel scaffolds may be better suited to support odontogenic differentiation of DPSCs than standard osteo/odontogenic medium or Col-I hydrogel scaffolds.

### Odontogenic supplements enhance the intrinsic odontogenic potential of bECM hydrogel scaffolds by further upregulating the odonto-specific genes expression of DPSCs

**Fig 4:**
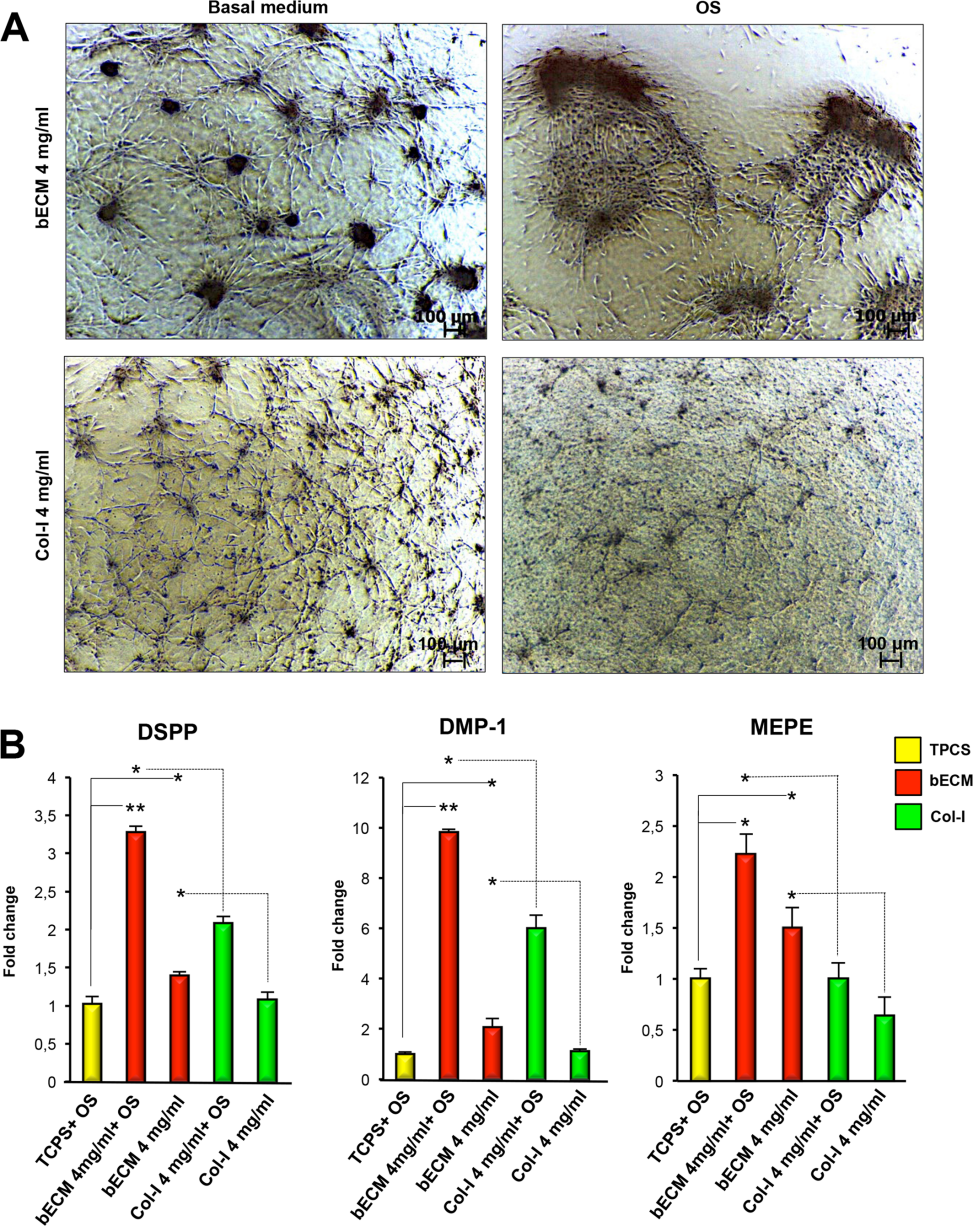
Morphology and odontogenic gene expression of DPSCs on bECM or Col-I hydrogel scaffolds cultured in medium containing odontogenic inducers. (A) Representative images of human DPSCs cultured on 4 mg/ml bECM or 4 mg/ml Col-I hydrogel scaffolds in osteo/odontogenic medium (OM) for 3 weeks, scale bar: 100 μm. (B) qRT-PCR at 3 weeks for odontogenic markers DSPP, DMP-1 and MEPE of DPSCs seeded on 4 mg/ml bECM hydrogel scaffold, 4 mg/ml Col-I or TCPS cultured in osteo/odontogenic medium. The results are presented as the fold increase (2^-(ΔΔCT)^) with respect to the level expressed in DPSCs seeded on TCPS cultured in osteo/odontogenic medium. The data shown are the means ± SDs of three independent experiments, *P < 0.05, ** *P* < 0.01 compared with DPSCs cultured osteo/odontogenic medium or 4 mg/ml bECM cultured in basal medium. OM: osteo/odontogenic medium.

To further evaluate the effect of the bECM hydrogel scaffolds on odontogenic differentiation of DPSCs, we performed qRT-PCR analysis for odontogenic-specific genes in DPSC cultures differentiated for three weeks on 4 mg/ml bECM and Col-I hydrogel scaffolds in osteo/odontogenic medium (Fig 4A). DPSCs grown on TCPS cultured in osteo/odontogenic medium, and on 4 mg/ml bECM or Col-I hydrogel scaffold cultured in basal medium were used as controls. qRT-PCR analysis demonstrated that the expression levels of DSPP, DMP-1 and MEPE were significantly higher in DPSCs seeded on bECM hydrogel scaffolds and cultured with osteo/odontogenic medium compared with those cultured in basal medium (Fig 4B). More importantly, the mRNA expression levels of DSPP, DMP-1 and MEPE were considerably higher in DPSCs seeded on 4 mg/ml bECM hydrogel scaffold and cultured in odontogenic media compared to those seeded on 4 mg/ml Col-I hydrogel scaffold or on TCPS cultured in osteo/odontogenic medium.

### Growth factors enhance the odontogenic differentiation potential of bECM hydrogel scaffolds

**Fig 5:**
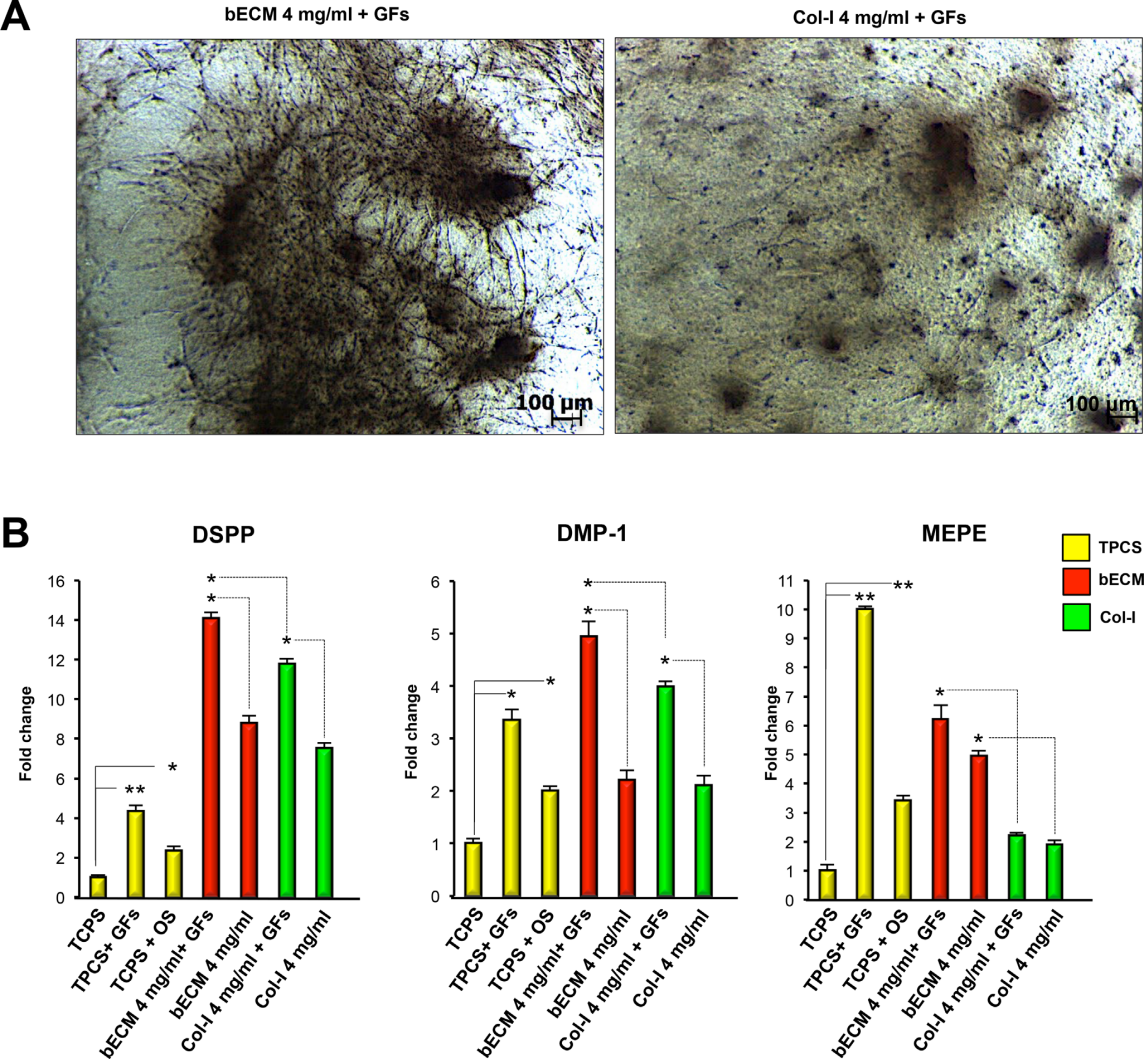
Distribution and odontogenic gene expression of DPSCs on bECM or Col-I scaffolds cultured in medium containing growth factors (GFs). (A) Representative images of human DPSCs cultured on bECM scaffolds (4 mg/ml) and Col-I (4 mg/ml) in medium containing GFs (FGFb + EGF) for 3 weeks, scale bar: 100 μm. (B) qRT-PCR at 3 weeks for odontogenic markers DSPP, DMP-1 and MEPE of DPSCs seeded on 4 mg/ml bECM or 4 mg/ml Col-I hydrogels cultured in medium containing GFs (FGFb + EGF) compared to those seeded on TCPS and cultured in basal or osteo/odontogenic medium (OM). The results are presented as the fold increase (2^-(ΔΔCT)^) with respect to the level expressed in DPSCs seeded on TCPS cultured in basal medium. The data shown are the means ± SDs of three independent experiments, *P < 0.05, ** *P* < 0.01 compared with TCPS-cultured cells in basal medium, osteo/odontogenic medium or seeded on 4 mg/ml bECM cultured in basal medium. OM: osteo/odontogenic medium, GFs: FGFb + EGF.

To evaluate the effect of FGFb + EGF on DPSCs cultured on bECM hydrogel scaffolds, we compared the odonto-specific gene expression of DPSCs seeded on 4 mg/ml bECM hydrogel scaffold in the presence or absence of GFs with respect to 4 mg/ml Col-I hydrogel scaffold and TCPS (Fig 5A) in the same culture conditions. DSPP, DMP-1 mRNA levels of DPSCs seeded on 4 mg/ml bECM hydrogel scaffold treated with GFs increased more than DPSCs on TCPS in the same conditions (Fig 5B). Conversely, MEPE mRNA levels of DPSCs seeded on TCPS and cultured in GFs medium expressed higher levels of MEPE compared to 4 mg/ml bECM hydrogel scaffold in the presence of GFs.

Importantly, compared to 4 mg/ml Col-I hydrogel scaffold in the presence of GFs, the DPSCs seeded on 4 mg/ml bECM hydrogel scaffolds and cultured with GFs showed a significant increase in the expression of DSPP, DMP-1 and MEPE genes (Fig 5B). These data establish that the odontogenic differentiation of DPSCs seeded on bECM hydrogels is also enhanced using medium supplemented with GFs.

## Discussion

Dental caries cause dentin-pulp tissue destruction that decreases the quality of life. The regeneration of dental tissues utilizing a scaffold-based tissue engineering approach represents a promising strategy to replace injured dental tissues and restore biological function [3, 36]. However, the realisation of dentin-pulp tissue regeneration depends on the use of appropriate scaffold materials that are able to stimulate the differentiation of the seeded dental stem/progenitor cells into tooth tissues. DPSCs are progenitor cells derived from adult human dental pulp that possess odontogenic abilities, providing a suitable cell source for dental tissue regeneration [4]. Several studies *in vitro* and *in vivo* have confirmed that DPSCs are one of the most promising candidates for dentin-pulp regeneration [15, 16, 29].

In this study, DPSCs extracted from human third molars and cultured on tissue culture polystyrene (TCPS) expressed membrane molecules CD13, CD29, CD44, CD73, CD90, CD105 and CD146, which confirmed the mesenchymal origin of the cells. The inherent odontogenic potential of the isolated DPSCs was also demonstrated.

However, when DPSCs are used for dentin-pulp tissue regeneration, it is crucial to enhance their odontogenic differentiation potential. This objective can be achieved by using appropriate scaffolds and growth factors. Recently, tissue-engineering-based strategies using both synthetic and natural scaffolds in combination with DPSCs have shown potential for the regeneration of the dentin-pulp complex [3, 29].

This study focuses on evaluating the odontogenic potential of hydrogel scaffolds derived from bone ECM (bECM) upon DPSCs. Here, we have used a demineralized and decellularized ECM derived from cancellous bovine bone to prepare the bECM hydrogel scaffolds [30–32].

We demonstrated that DPSCs seeded on bECM hydrogel scaffolds can undergo odontogenic differentiation without stimulus from external inducers or growth factors *in vitro*. In fact, we observed that bECM hydrogel scaffolds enabled upregulation of markers of odontogenic differentiation such as DSPP, DMP-1 and MEPE. These genes, encoded for non-collagenous proteins synthesized by odontoblasts, play a crucial role during early odontoblastic differentiation and late dentin mineralization [35, 37–40]. DSPP is the first putative marker of odontoblastic differentiation and its upregulation suggests that DPSCs may have been acquiring the capacity of secreting mineralizable dentin [41]. DMP-1 is an extracellular matrix glycoprotein important for the mineralization of dentin: during odontoblast maturation, DMP-1 is phosphorylated and exported to the extracellular matrix where it organizes formation of mineralized matrix [42, 43]. Matrix extracellular phosphoglycoprotein (MEPE) is a new member of bone matrix protein family that has been found in human bone and dental tissues [39, 40]. Importantly, Liu *et al*. showed that MEPE plays a regulatory role in the odontogenic induction of DPSCs [40].

Results from the qRT-PCR showed that the DPSCs could differentiate into odontogenic lineage when cultured on the bECM scaffolds. Furthermore, DPSCs cultured on bECM hydrogel scaffolds displayed higher odontogenic differentiation than those on TCPS in the presence of odontogenic inducers as indicated by qRT-PCR analysis. This finding is in line with recent observations that ceramic bovine bone scaffolds (CBB) possess significant odonto-inductive activity [29]. Our data demonstrated that compared to conventional Col-I hydrogels, the bECM hydrogels provide a better environment to support DPSCs odontogenic differentiation in basal culture conditions.

In the second part of this study, to further enhance the odontogenic differentiation of DPSCs induced by bECM, we used a combination of bECM with osteo/odontogenic medium. qRT-PCR analysis showed that the expression levels of DSPP, DMP-1 and MEPE were significantly higher in DPSCs seeded on 4 mg/ml bECM hydrogels cultured in osteo/odontogenic medium compared to culture in basal medium. More importantly, in DPSCs seeded on 4 mg/ml bECM hydrogel scaffolds the expression levels of DSPP, DMP-1 and MEPE were considerably higher compared to those seeded on Col-I hydrogel scaffolds or TCPS-cultured cells with osteo/odontogenic medium. The upregulated expression of odonto-related genes in bECM scaffolds treated with osteo/odontogenic medium suggests that DPSCs differentiation towards odontoblast-like cells is further enhanced in the presence of odontogenic supplements.

Growth factors may also be added to hydrogel scaffolds, and in fact, the addition of basic fibroblast growth factors (bFGF) within gelatin hydrogels induces the neovascularization and regeneration of tissues relevant to the dentin-pulp complex [44, 45]. bFGF, known also as FGF2, has been associated with tooth morphogenesis by contributing to the mineralization of dentin matrix [46]. Additionally, FGF-2 is able to upregulate *in vitro* the expression of DSP and DMP1 in human DPSCs [47]. Moreover, *Kim et al*. showed that FGF2 promoted odontoblastic differentiation of human DPSCs in *vitro* as evidenced by the formation of mineralized nodules and the upregulation of ondontoblast-specific genes such as DSPP, DMP-1 and MEPE [48].

In addition, Bonnamain *et al*. showed that DPSCs induced to form spheroid structures in medium containing EGF were able to upregulate the expression of the odonto-specific gene DSPP *in vitro* [49].

In our study, using a combination of GFs containing bFGF and EGF, we observed odontogenic differentiation of DPSCs cultured on 4 mg/ml bECM with upregulation of DSPP, DMP1 and MEPE mRNA levels respect to DPSCs on TCPS or 4 mg/ml Col-I-in the same conditions. Taken together, these results also demonstrate that the combination of bECM hydrogel scaffolds with GFs is a suitable method for the enhancement of odontogenic differentiation of DPSCs cultured *in vitro*.

Overall, this study demonstrates that bECM hydrogel scaffolds are capable of inducing odontogenic differentiation of DPSCs *in vitro*. This study also shows that it is possible to achieve bECM mediated odontogenic differentiation of DPSCs without the need for odontogenic media or growth factors. However, the use of these inducers in combination with bECM scaffolds provides a higher potential of odontogenic differentiation of DPSCs.

## Conclusions

Although many strategies to regenerate pulp/dentin have been considered [3, 18–22], the generation of functional dentin and effective odontoblasts has not yet been achieved. Our *in vitro* data show that the bECM hydrogel scaffolds can be used to promote odontogenic differentiation of DPSCs in the absence of external inducers and can be combined with osteo/odontogenic medium or growth factors to further enhance this intrinsic odontogenic potential. Furthermore, bECM hydrogel scaffolds appear to support odontogenic differentiation of DPSCs more effectively than Col-I hydrogel scaffolds cultured at the same conditions. Consequently, the bECM hydrogel scaffolds offer an excellent environment for odontogenic differentiation of DPSCs and are encouraging candidates for dentin-pulp regeneration.

These findings represent the initial step for the development of a novel class of scaffolds able to induce odontogenic differentiation of DPSCs. Further analysis will be necessary to verify the suitability of these scaffolds for odontogenic differentiation of DPSCs in *vivo*. Further studies will utilize a rat pulp injury model to evaluate mineralized dentin formation produced by DPSCs/bECM constructs.

## Supporting Information

S1 Fig Morphology and distribution of DPSCs seeded or encapsulated in bECM hydrogels. DPSCs were seeded on 4 mg/ml bECM hydrogel or encapsulated in 4 mg/ml bECM hydrogel and morphology and distribution assessed after 2 weeks. (A) Macroscopic view of DPSCs/scaffold constructs. (B) Contrast phase images of DPSCs seeded or encapsulated in bECM hydrogels. Scale bar, 100 μm.
(TIF)

S2 Fig bECM supports cell viability during initial cell seeding. DPSCs were seeded on 4 mg/ml bECM hydrogel (A) or encapsulated in 4 mg/ml bECM hydrogel (B) and viability assessed after 15 minutes, 1h, 2h, and 12h using a live/dead staining. Scale bar, 100 μm.
(TIF)

S3 Fig Von Kossa staining views of DPSCs/scaffold constructs. DPSCs were cultured on 4 mg/ml bECM and Col-I hydrogel scaffolds in basal medium for 3 weeks. Black color indicates mineral deposition. Scale bar: 100 μm.
(TIF)
