# Supplementary figures and images for: Odontogenic Differentiation of Human Dental Pulp Stem Cells on Hydrogel Scaffolds Derived from Decellularized Bone Extracellular Matrix and Collagen Type I
Source: PLoS One. 2016 Feb 16;11(2):e0148225. doi: 10.1371/journal.pone.0148225 (PMC4755593; doi:10.1371/journal.pone.0148225)

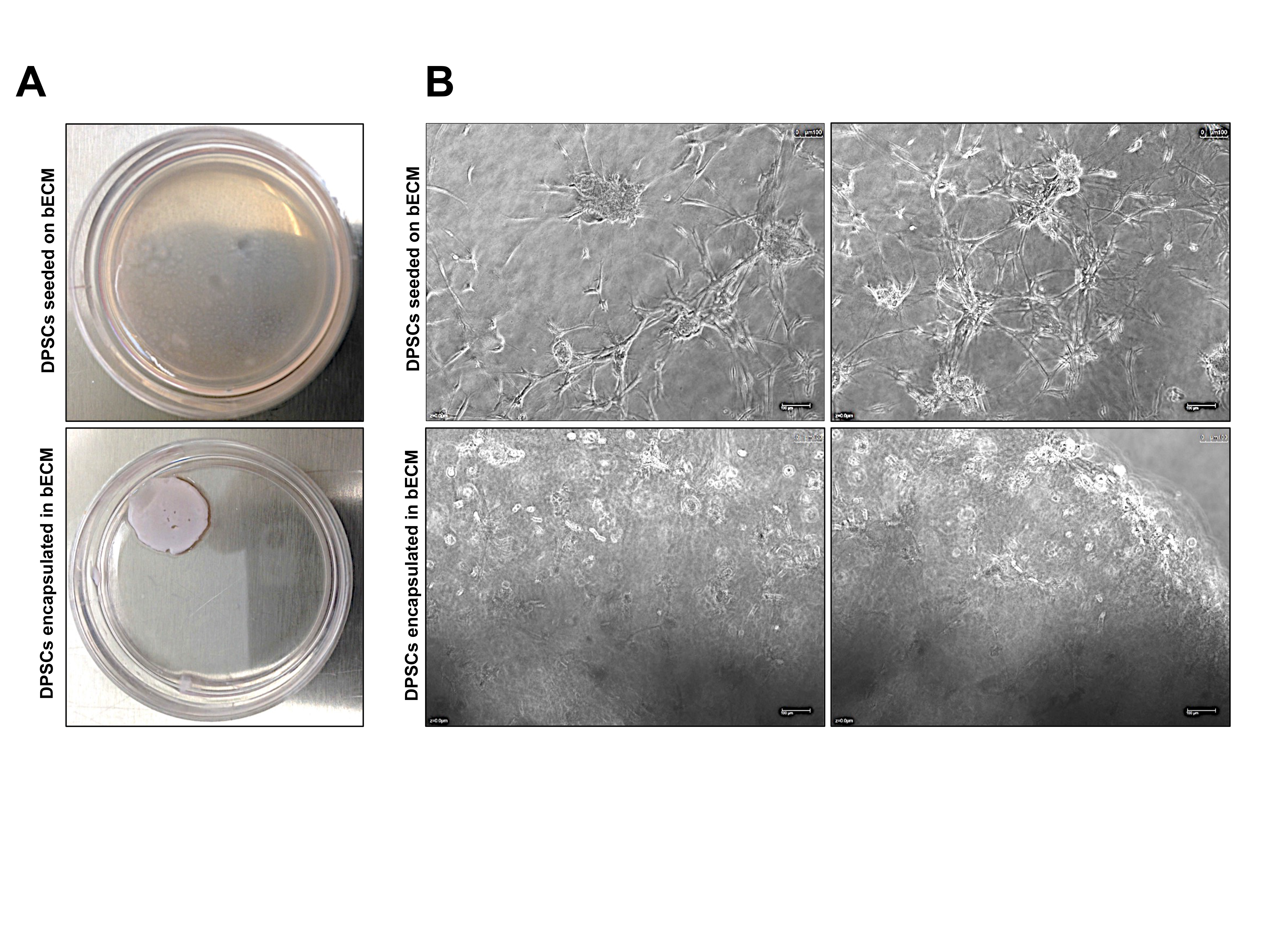

Supplement: S1 Fig — DPSCs were seeded on 4 mg/ml bECM hydrogel or encapsulated in 4 mg/ml bECM hydrogel and morphology and distribution assessed after 2 weeks. (A) Macroscopic view of DPSCs/scaffold constructs. (B) Contrast phase images of DPSCs seeded or encapsulated in bECM hydrogels. Scale bar, 100 μm. (TIF) [file pone.0148225.s001.tif]

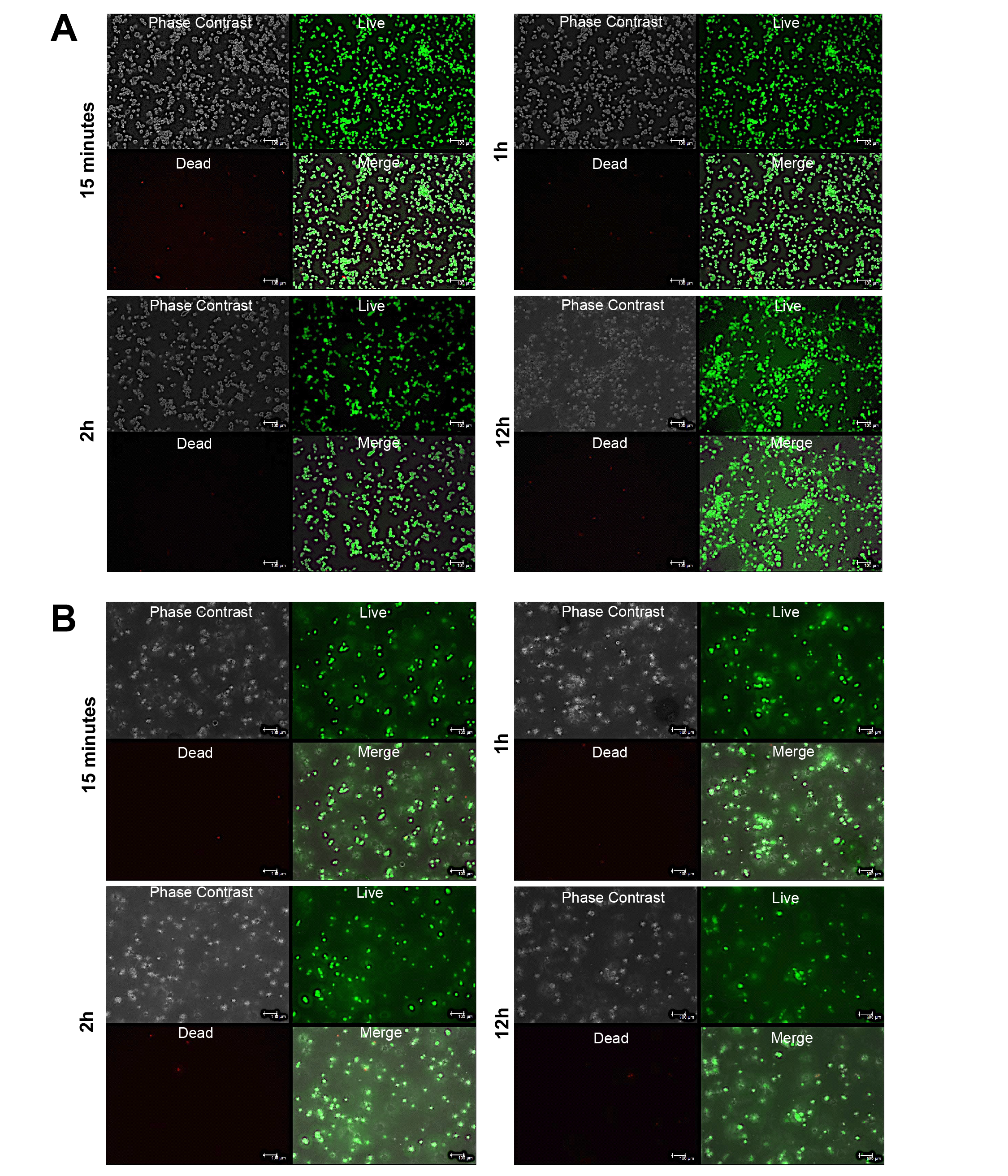

Supplement: S2 Fig — DPSCs were seeded on 4 mg/ml bECM hydrogel (A) or encapsulated in 4 mg/ml bECM hydrogel (B) and viability assessed after 15 minutes, 1h, 2h, and 12h using a live/dead staining. Scale bar, 100 μm. (TIF) [file pone.0148225.s002.tif]

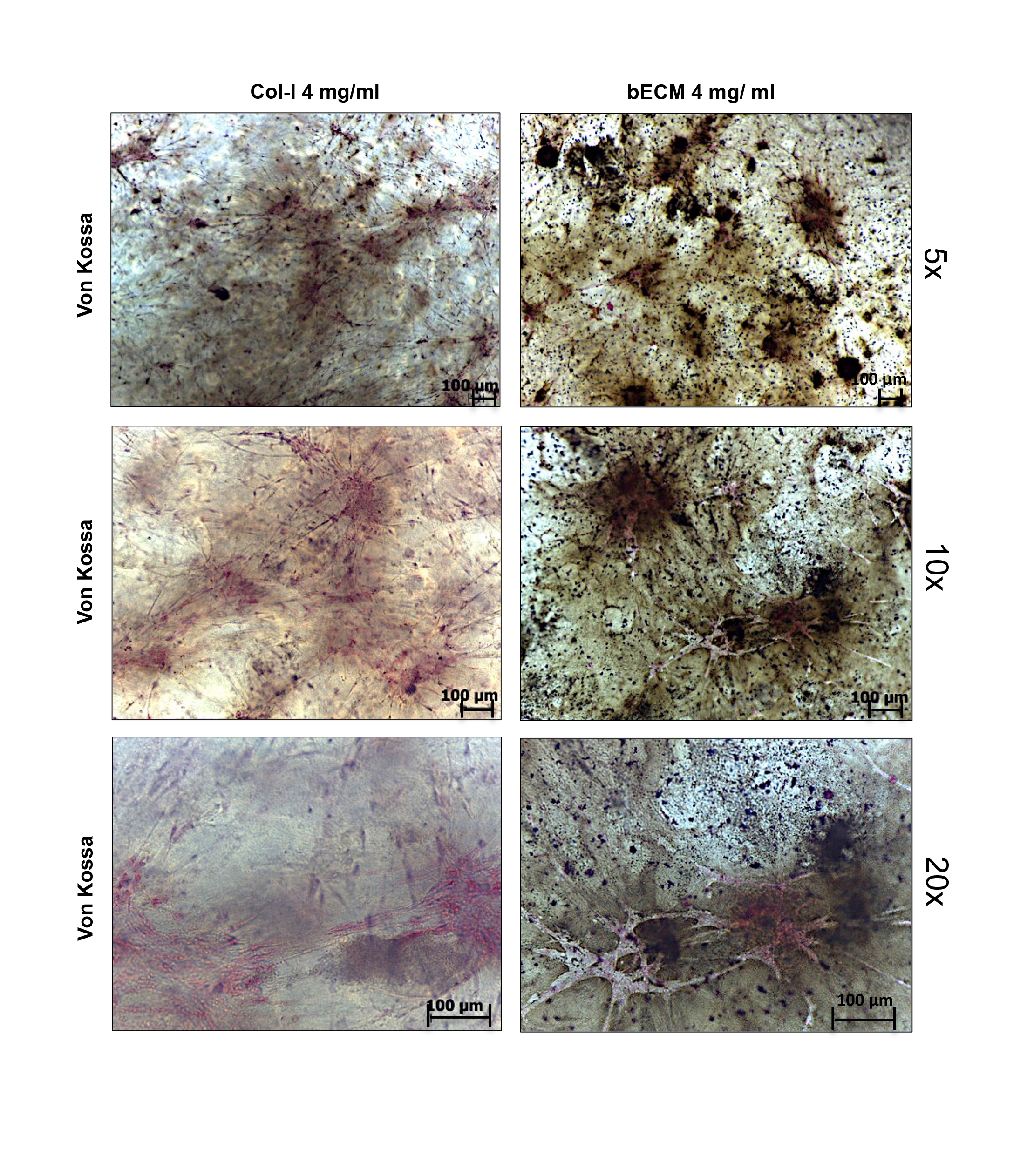

Supplement: S3 Fig — DPSCs were cultured on 4 mg/ml bECM and Col-I hydrogel scaffolds in basal medium for 3 weeks. Black color indicates mineral deposition. Scale bar: 100 μm. (TIF) [file pone.0148225.s003.tif]
